# Prediction model for severe vesicoureteral reflux in children with urinary tract infection and/or hydronephrosis

**DOI:** 10.1007/s00467-025-06668-7

**Published:** 2025-01-21

**Authors:** Pelin Laleoğlu, Gizem Yildiz, Meral Torun Bayram, Handan Güleryüz Uçar, Salih Kavukcu, Alper Soylu

**Affiliations:** 1 Department of Pediatrics, Dokuz Eylül University Medical Faculty, Balçova, İzmir, 35340 Turkey; 2 Department of Pediatric Nephrology, Dokuz Eylül University Medical Faculty, Izmir, Turkey; 3 Department of Radiology, Dokuz Eylül University Medical Faculty, Izmir, Turkey; 4 Department of Pediatric Nephrology and Rheumatology, Dokuz Eylül University Medical Faculty, Izmir, Turkey

**Keywords:** Severe vesicoureteral reflux, Voiding cystourethrography, Urinary tract infection, Non-*E.coli* uropathogen, Kidney scar, Prediction model

## Abstract

**Background:**

As voiding cystourethrography is invasive and exposes to radiation and urinary tract infection (UTI), identifying only high-grade reflux is important. We aimed to identify clinical, laboratory and imaging variables associated with high-grade primary reflux in children presenting with UTIs and/or urinary tract dilatation and develop a prediction model for severe reflux.

**Methods:**

Data of children who underwent voiding cystourethrography due to UTI and/or urinary tract dilatation were retrospectively analyzed for demographic, clinical and imaging findings. Patients with severe (grades 4–5) reflux were compared with the rest for these parameters and a prediction model was developed for severe reflux.

**Results:**

The study included 1044 patients (574 female). Severe reflux was present in 86 (8.2%) patients. Age < 2 years, male sex, non-*E. coli* uropathogens, UTD-P3 dilatation and multiple kidney scars on DMSA scintigraphy were associated with severe reflux. Using these variables a prediction model for severe reflux with a score ranging from 0–7 and accuracy rate of 93.4% was developed. A score ≥ 5 had sensitivity 44.2%, specificity 97.4%, PPV 60.3%, NPV 95.1% and OR 29.5 for severe reflux. Scores ≥ 5 and ≥ 4 catch 44% and 73% of severe reflux, while prevent invasive voiding cystourethrography in 94.0% and 83.6% of patients, respectively.

**Conclusion:**

Age < 2 years, male sex, non-*E. coli* uropathogen growth, presence of UTD-P3 dilatation on ultrasonography and multiple scars on DMSA scintigraphy are risk factors for severe reflux. A scoring system based on these variables appears to be effective in predicting the presence of severe reflux and eliminating unnecessary voiding cystourethrography.

**Graphical abstract:**

**Figure:**
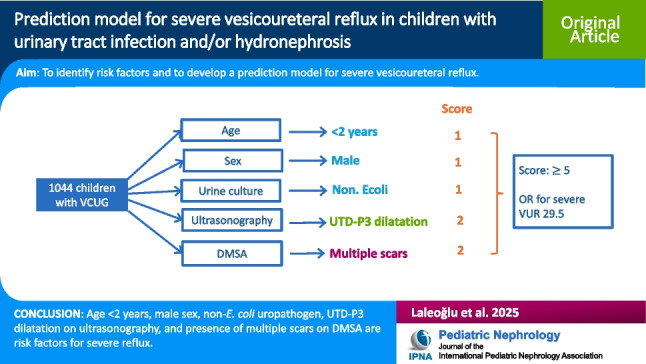
A higher resolution version of the Graphical abstract is available as Supplementary information

**Supplementary Information:**

The online version contains supplementary material available at 10.1007/s00467-025-06668-7.

## Introduction

Vesicoureteral reflux (VUR) is an anatomical and functional disorder characterized by the abnormal backward flow of urine from the bladder into the ureters and renal pelvis. Primary VUR is a common urological anomaly in children. One third of children with urinary tract infections (UTIs) have VUR. Although VUR by itself is not a cause of kidney damage postnatally, if associated with recurrent infections it can cause kidney scarring in 10–40% of children which poses a risk of causing serious complications such as kidney failure, and the prevention of kidney damage forms the basis of treatment [1]. Historically, the detection of all VUR cases was recommended to prevent chronic kidney disease, hypertension, and kidney failure caused by UTIs. However, the use of voiding cystourethrography (VCUG) for diagnosing VUR requires urethral catheterization with the risk of iatrogenic UTIs and exposes to radiation [2]. On the other hand, recent publications report that kidney failure caused by reflux nephropathy is extremely rare (< 0.0005%) [3]. Most cases of primary VUR are low grade (grades 1–3) and have a low likelihood of being associated with acquired kidney scarring [1, 2]. Conversely, children diagnosed with high-grade VUR (grades 4–5) are at the greatest risk for UTIs and kidney scarring during infancy [3]. While spontaneous resolution of low grade VUR is fast, only 30–50% of high grade VUR resolves within 4–5 years. Thus, children with grades 4–5 VUR have higher risk of kidney scarring and associated complications. Early identification of these children necessitates conservative approaches, like circumcision in early infancy, prophylactic antibiotics and surgical intervention in case of breakthrough UTIs despite these measures [4]. Therefore, identifying high-grade VUR has gained importance, and VCUG should be requested only in selected patients. On the other hand, high-grade reflux detected mostly in males presenting with antenatal hydronephrosis/renal parenchymal defects is associated with congenital dysplasia with inevitable renal functional deterioration later in life [5, 6].

In this study, we aimed to identify clinical, laboratory and imaging variables that could be helpful in predicting high-grade primary reflux in children presenting with UTIs and/or urinary tract dilatation and to develop a prediction model for grades 4–5 reflux regardless of its clinical significance.

## Materials and methods

### Patients

Data from 1044 patients who underwent VCUG due to UTI and/or urinary tract dilatation at Dokuz Eylul University Pediatric Nephrology Clinic between 2010 and 2020 were retrospectively analyzed.

This study was conducted with the approval of the Non-Interventional Ethics Committee of Dokuz Eylul University School of Medicine, under the code 2023/23–13.

## Methods

The files of the patients were evaluated for age, gender, presence of UTI, causative microorganism, urinary system ultrasonography (USG), Tc-99 m dimercaptosuccinic acid (DMSA) renal scintigraphy and VCUG results, retrospectively. As we aimed to evaluate the parameters associated with primary high-grade VUR, children with secondary VUR (bladder bowel dysfunction, obstructive uropathy like posterior urethral valve, double collecting system with ureterocele, etc.) were excluded.

### Urinary tract infections

UTI was defined as growth of a single microorganism over 100,000 col/mL in association with fever and/or specific symptoms related to UTIs. Recurrent UTI was defined as two or more episodes of UTI. Uropathogens were grouped as *E. coli* and non-*E. coli*.

### Ultrasonography

All patients underwent urinary system USG. USG results were classified as normal vs. abnormal (dilatation, abnormal parenchyma, calculi), presence vs. absence of hydronephrosis (HN), and the degree of HN. The degree of HN was determined according to the international urinary tract dilatation system as UTD-P 1–3 [7].

### VCUG

VCUG was performed in children with UTI in the presence any of the following features: complicated UTI, recurrent UTI, abnormal ultrasonography findings, non-*E. coli* infections, or abnormal DMSA findings. In children with antenatal HN, VCUG was performed in the presence of symptomatic UTI, UTD-P2 with dilated ureter or UTD-P3 [6]. VUR was classified according to the criteria of the International Reflux Study Group [8]. In patients with bilateral reflux, the highest degree of VUR was recorded. Grades 4–5 were defined as severe VUR.

### Tc-99m DMSA scintigraphy

Imaging results were evaluated in terms of the presence of any scar and multiple scars. Kidneys with cortical defects and/or irregular margins were considered to have scarring. Decreased kidney function was defined as relative uptake less than 45% [9].

## Statistical analyses

Variables associated with severe VUR were identified using the chi-square test. We used the odds ratios from the chi-square test for the severe VUR prediction model. Scoring for each variable was determined using each OR divided by the lowest OR to simplify the scoring system. To determine cut-off scores and evaluate the accuracy of the model, ROC (Receiver Operating Characteristic) analysis was performed. Cut-off scores were calculated using sensitivity, specificity values, and Youden's Index. The area under curve (AUC) was provided with 95% confidence intervals (CI). A p-value of less than 0.05 was considered statistically significant. All statistical analyses were conducted using SPSS version 29.

## Results

Between 2010 and 2020, a total of 1044 patients who underwent VCUG and met our study criteria were included, comprising 574 (55.0%) females and 470 (45.0%) males. The mean age of the patients at the time of VCUG was 29.0 ± 35.1 months (median 12, range 0–167 months).

Severe VUR was present in 86 (8.2%) patients in our study population. Patients with severe VUR were younger (19.1 ± 27.6 vs. 29.9 ± 35.5 months, p > 0001). Demographic and clinical characteristics of the patients with severe VUR vs. no/mild VUR are presented in Table 1. Patients with severe VUR were more commonly male and less than 2 years old. A history of UTIs and the number of UTIs were not associated with the frequency of severe VUR; however, the presence of non-*E. coli* uropathogens in culture was associated with severe VUR. Pathological USG findings, HN, and particularly UTD-P3 HN were more frequently observed in children with severe VUR. DMSA scintigraphy was performed on 507 patients. The presence of any scar and multiple scars on DMSA was also more common in children with severe VUR. Thus, the variables associated with severe VUR included age < 2 years, male sex, presence of non-*E. coli* uropathogens in culture, abnormal USG (any abnormality, HN and UTD-P3 HN), and abnormal DMSA (any scar and multiple scars).

**Table 1:** Comparison of the children with severe vesicoureteral reflux (VUR) and those with no/mild VUR

Parameters	Children who underwent VCUG (n = 1044)		*p*	OR (%95 CI)	Score
	No/mild VUR (*n* = 958)	Severe VUR (*n* = 86)			
Age < 2 years	610 (63.7)	65 (75.6)	**0.027**	**1.8 (1.1–2.9)**	**1**
Male, n (%)	416 (43.4)	54 (62.8)	** < 0.001**	**2.2 (1.4–3.5)**	**1**
UTI, n (%)	559 (58.3)	58 (67.4)	0.100		
Recurrent UTIs, n (%)	354 (36.9)	35 (40.7)	0.117		
Non-*E.Coli*, n (%)	206 (21.5)	32 (37.2)	**0.006**	**2.6 (1.7–4.1)**	**1**
Abnormal USG, n (%)	650 (67.8)	73 (84.9)	0.001	2.7 (1.5–4.9)	
Hydronephrosis, n (%)	439 (45.8)	68 (79.1)	< 0.001	4.5 (2.6–7.6)	
UTD-P3 hydronephrosis, n (%)	138 (14.4)	43 (50.0)	** < 0.001**	**5.9 (3.8–9.4)**	**2**
Scarring*, n (%)	242 (55.1)	56 (82.4)	< 0.001	3.8 (2.0–7.3)	
Multiple scarring*, n (%)	147 (33.5)	50 (73.5)	** < 0.001**	**5.5 (3.1–9.8)**	**2**

*USG:* Ultrasonography*, UTD:* Urinary tract dilatation*, UTI:* Urinary tract infection*, VCUG:* Voiding cystourethrography

^*^DMSA scintigraphy was performed in 507 patients

**Fig. 1:**
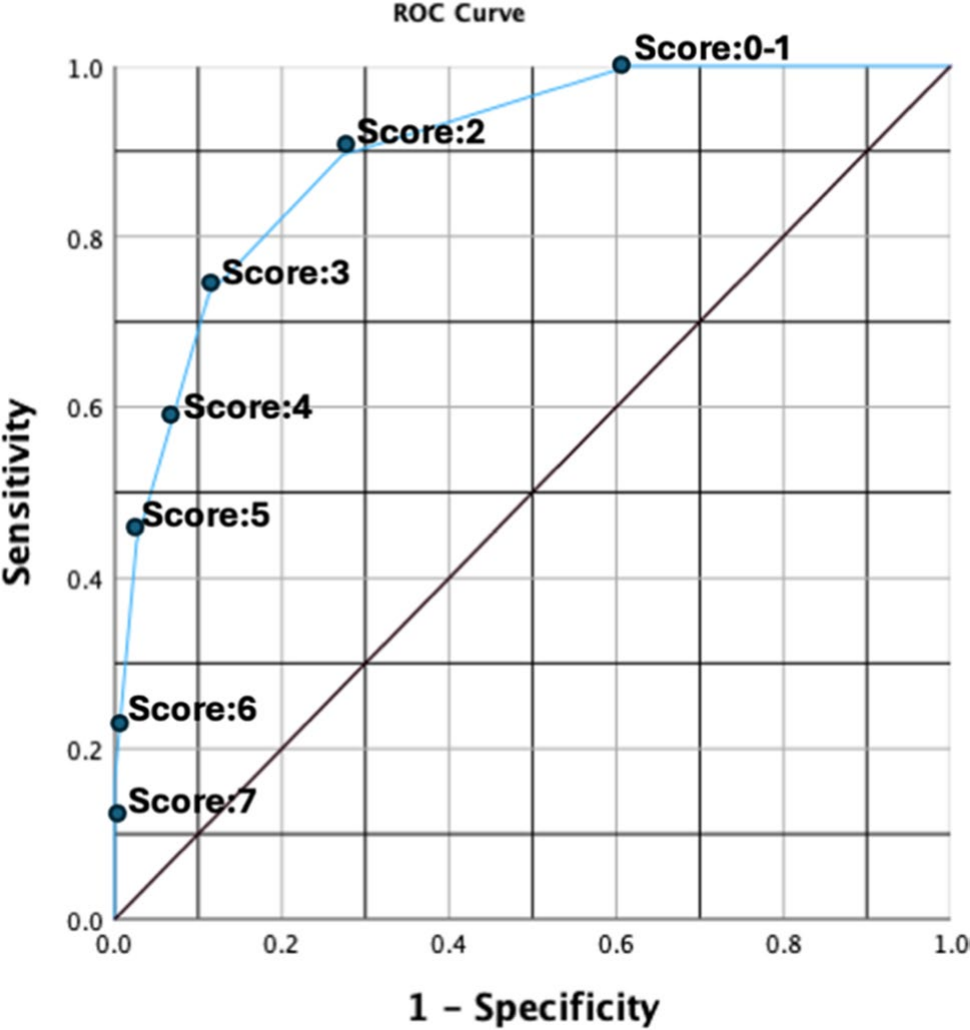
ROC curve showing the sensitivity and specificity of different scores for predicting severe (grades 4–5) vesicoureteral reflux (low risk: 0–2 points; moderate risk: 3–4 points; high risk: 5–7 points)

A prediction model for severe VUR was developed using ORs of the relevant variables. However, as variables related to USG and DMSA are interrelated, only one ultrasonographic (UTD-P3 HN) and one scintigraphic (multiple scars) variable with highest OR were selected. Each relevant variable was approximately weighted according to the OR and assigned scores of 1 or 2 (Table 1). Based on these scores, the calculated scores ranged from a minimum of 0 to a maximum of 7 in predicting severe VUR. The observed rates of severe VUR according to the patients' scores are presented in Table 2. The AUC from the ROC analysis was found to be 0.901, with a 95% confidence interval (CI) ranging from 0.873 to 0.929 (Fig. 1). Cut-off scores calculated using Youden’s Index were determined as follows: low risk: 0–2; moderate risk: 3–4; high risk: 5–7. In the low risk group, severe VUR was quite rare (1.2%), while in the moderate risk group, this rate was 14.2%, and in the high-risk group, the presence of severe VUR was 60.3%. The difference between these groups was significant (Table 3). Among the 981 patients with a score ≤ 4, 48 (4.9%) had severe VUR, whereas among the 63 patients with a score ≥ 5, 38 (60.3%) had severe VUR. According to these parameters, a score ≥ 5 had a sensitivity 44.2%, specificity 97.4%, positive predictive value (PPV) 60.3%, and negative predictive value (NPV) 95.1% for severe VUR. Patients with a score ≥ 5 were 29.5 (95% CI: 16.6–52.9) times more likely to have severe VUR compared to those with a score ≤ 4 (Table 4). Additionally, none of the patients with a score ≤ 1 had severe VUR, and the rate of severe VUR among those with a score 2 was 2.7%. In contrast, among patients with a score 6 or 7, 100% had severe VUR. A score ≥ 5 detects 38 severe VUR in 63 patients (60.3%) eliminating invasive VCUG in 981 patients. However, we can catch only 44.2% of all severe VUR. If VCUG was performed in children with a score ≥ 4, 63 severe VUR cases are detected in 171 patients (36.8%). This corresponds to 73.2% of all severe VUR cases, eliminating unnecessary VCUG in 873 children.

**Table 2:** Patients’ scores and severe (grade 4–5) vesicoureteral reflux (VUR) rates according to the “severe VUR prediction model”

Total score	Number of patients		Severe VUR %
	Severe VUR (-)	Severe VUR ( +)	
7	-	1	100.0
6	-	13	100.0
5	25	24	49.0
4	83	25	23.1
3	153	14	8.4
2	329	9	2.7
1	228	-	0.0
0	140	-	0.0

**Table 3:** Comparison of low, moderate and high-risk patients for the presence of severe vesicoureteral reflux (VUR)

Cut-off scores	Severe (grades 4–5) VUR		*p*
	Absent *n* (%)	Present *n* (%)	
Low risk (0–2)	697 (98.8)	9 (1.2)	< 0.001
Moderate risk (3–4)	236 (85.8)	39 (14.2)	
High risk (5–7)	25 (39.7)	38 (60.3)

**Table 4:** Predictive power of our model for severe (grades 4–5) vesicoureteral reflux

Cut-off scores	Diagnostic performance				
	Sensitivity	Specificity (%)	PPV (%)	NPV (%)	Odds ratio
High risk (5–7)	44.2	97.4	60.3	95.1	29.5 (16.6–52.9)
Moderate risk (3–4)	45.3	75.4	14.2	93.9	2.5 (1.6–4.0)
Low risk (0–2)	10.5	27.2	1.3	77.2	0.04 (0.02–0.09)

*PPV:* Positive predictive value*, NPV:* Negative predictive value

DMSA scintigraphy was not performed in 537 patients. Thus, the prediction model in those patients is based on a maximum score of 5. When we evaluated these patients separately, only 18 (3.3%) had severe VUR and half of them (9 patients) had a score 5. The rate of severe VUR among children with a score 5 was 37.5% (9/24), while it was only 1.8% (9/513) in children with a score ≤ 4 (p < 0.001). Sensitivity, specificity, PPV, NPV and OR of score 5 for predicting severe VUR were 50.0%, 97.1%, 37.5%, 98.2% and 33.6, respectively.

## Discussion

The prevalence of VUR is around 30% in children with a history of UTI and 10–20% in newborns with antenatal HN. On the other hand, up to 30% of patients with antenatal HN may develop UTIs, and the risk of infection correlates with the grade of dilatation. VCUG is recommended by the American Urological Association and European Association of Urology in children with antenatal HN that develop UTI [6]. Thus, a substantial number of children with antenatal HN develop UTIs on follow-up, while HN might be determined during the evaluation of children with UTI. Since it is difficult to evaluate antenatal HN and UTI separately in a retrospective study, we included both variables in the same scoring system.

VCUG is indicated in children with UTIs and in infants with pelvicalyceal dilatation [6]. Infants with antenatal HN and high-grade VUR may have congenital renal dysplasia and also have the potential for ongoing renal developmental impairment due to high pressure sterile reflux especially in males with abnormal bladder dynamics. In the presence of bladder dysfunction and DMSA abnormalities, VUR resolution is less likely and breakthrough UTIs are more common [5]. As the risk of renal damage increases in direct proportion to the severity of VUR [6], early identification of infants with high-grade VUR with ongoing risk of renal functional deterioration is important for appropriate management. Thus, we specifically aimed to detect the predictors of high-grade VUR.

Many guidelines published on the management of UTIs in children have narrowed the indications for VCUG, emphasizing that it should be performed only in selected patients [10–13]. Numerous studies have shown that a history of UTI in children is associated with high-grade VUR [14–17]. Contrary to the literature, our study did not find an association between a history of UTIs or the number of UTIs and the frequency of severe VUR. Our study population included both children presenting with UTIs and those presenting with antenatal HN. Almost half of the children (507/1044) had some degree of hydronephrosis. Furthermore, half of the children with high-grade VUR (43/86) had UTD-P3 hydronephrosis and almost two thirds of these children were male with globally decreased kidney function (Table 1). This indicates that most children with grades 4–5 VUR were males having congenital renal dysplasia with a lower risk of UTI. This could explain the lack of difference in UTIs between the groups. On the other hand, the frequency of severe VUR was higher in patients with non-*E. coli* uropathogens (Table 1). Similarly, a study examining 738 patients aged 0–24 months with a history of UTIs found a higher rate of VUR in those with non-*E. coli* uropathogens compared to those with *E. coli* [18]. Another retrospective study reported that the presence of non-*E. coli* uropathogens in cultures was the only significant predictor of severe VUR [16]. Therefore, it is more important to perform VCUG in patients with non-*E. coli* UTIs rather than in all patients with a history of UTIs. This approach is consistent with many current guidelines [12, 19, 20]. However, only 32 of 238 (13.4%) children with non-*E. coli* infection had severe VUR in the present study. These 32 children had significantly higher rate of UTD-P3 HN and multiple kidney scars compared to the other 206 children. Among these 32 children 14 (43.8%) had UTD-P3 HN and 12 (38.0%) had multiple scars in DMSA scintigraphy. Thus, although the score of non-*E. coli* infection is only 1 by itself, total scores of these 32 children were 2 (1 case), 3 (6 cases), 4 (8 cases), 5 (14 cases), 6 (2 cases), and 7 (1 case). This means 17 of 32 (53.1%) children were in high-risk group and 25 (78.1%) had a score ≥ 4. These data suggest that, even in the presence of non-*E.coli* infections, clinicians' decision to perform VCUG by taking other parameters into consideration will increase the detection rate of high-grade VUR. Thus, our scoring system integrating the results of imaging techniques contributes significantly to daily clinical practice.

The selection of imaging tests following UTIs in children remains a contentious issue. Many clinical guidelines recommend performing USG for all patients after a UTI [12, 21, 22]. However, USG results are normal in over 80% of cases, leading to significant time and cost inefficiencies [23–25]. Despite the limited sensitivity of USG in detecting VUR, most clinical guidelines recommend VCUG for abnormalities detected on USG [24, 26]. In our study, the rate of severe VUR was increased in the presence of any abnormality on USG, particularly UTD-P3 HN (Table 1). Nonetheless, numerous studies have shown that USG alone is insufficient for diagnosing VUR [14, 27–32].

The approach of performing DMSA imaging first and then VCUG after a febrile UTI is known as the "top-down approach". The top-down approach assumes that clinically significant VUR will have associated changes on DMSA scan and that VUR is clinically insignificant in patients with normal DMSA scans [33]. In a prospective study evaluating the top-down approach, 85% of patients with abnormal DMSA had high-grade VUR [34]. A Cochrane review found that children with negative DMSA scans had less than 1% chance of having high-grade VUR [35]. Similarly, various studies have shown that children with severe VUR are more likely to develop kidney scarring [28, 36]. In our study, the presence of multiple scars on DMSA was associated with an increased frequency of severe reflux (Table 1). However, it should be kept in mind that abnormalities in DMSA scintigraphy may be due to either congenital dysplasia or acquired postinfectious damage. Congenital kidney injury is specific to high-grade VUR and seen mostly in males [5]. This kind of VUR may be detected upon evaluation of children with antenatal hydronephrosis or renal parenchymal defects [6]. These children are more prone to inevitable renal functional impairment later in life [5].

In a study using data from 324 children aged 2–60 months with a history of febrile UTI, a scoring system was developed, and independent risk factors for high-grade VUR were identified as recurrent UTIs, non-*E. coli* pathogens, and abnormal USG findings [37]. Similarly, in our model, age < 2 years, male sex, infection by non-*E. coli* uropathogens, the presence UTD-P3 HN on USG and multiple scars on DMSA were also related to severe VUR.

In our model, the total score ranges from 0 to 7. The cut-off scores were determined as follows: low risk 0–2, moderate risk 3–4, high risk 5–7. Given that a score ≥ 5 has very high specificity for severe VUR, it may be considered to avoid performing VCUG in patients with lower scores.

Guidelines established by major institutions such as the American Academy of Pediatrics and the National Institute for Healthcare and Excellence have significantly reduced the number of unnecessary VCUGs [10, 38]. However, these guidelines have also led to delays in the diagnosis and treatment of some children with VUR. Various studies reviewing these guidelines have shown that most of the missed cases involve children with low grade VUR. Even if children with low grade VUR are not diagnosed after the first UTI, the impact on patients is minimal because antimicrobial prophylaxis is not routinely recommended for these children [19, 20, 39, 40]. The key is not to diagnose every case of VUR but to identify and treat other risk factors for UTIs, such as bladder and bowel dysfunction [41, 42].

According to our prediction model for severe VUR, patients in the low-risk group (score 0–2) constituted 67.6% of all patients, and the rate of severe VUR in this group was 1.2%. Therefore, VCUG should not be performed in patients within the low-risk group. In the moderate-risk group (score 3–4), although the frequency of severe VUR was only 10.9%, one third of all severe VUR cases (29/86) were in this group. Thus, the decision to perform VCUG in this group should be carefully evaluated by clinicians. In the high-risk group (score 5–7), the rate of severe VUR was 60.3%. Clinicians should not hesitate to request VCUG for patients in this high-risk group.

Almost all clinical guidelines recommend VCUG in children with UTIs in the presence of any of the following features: complicated UTI, recurrent UTI, non-*E. coli* infection, abnormal ultrasonography findings [6]. In fact, we also performed VCUG according to these guidelines. However, severe VUR was only present in 8.2% of children. The present study, based on a scoring system integrating all these variables along with DMSA findings, implies that a score ≥ 5 is almost 30 times more likely to reveal severe VUR. Performing VCUG in children with a score ≥ 5 will yield severe VUR in almost half of these children at the expense of overlooking half of the severe VUR in the remaining children. Thus, sensitivity of this scoring system is low. However, as its specificity is high, by doing this, unnecessary VCUG would be avoided in 94% (981/1044) of children.

Limitations of our study include retrospective design which might have precluded the full differentiation between cystitis and pyelonephritis due to incomplete clinical and laboratory data on the patient files. In addition, information on the method of urine culture collection was not available in most patient records, so instances of growth below 100,000 colonies in samples obtained by urinary catheterization may have been missed. Furthermore, approximately half of the patients included in the study underwent DMSA imaging. Patients without a DMSA scan could achieve a maximum score 5. However, evaluation of children without DMSA scintigraphy also showed that probability of severe VUR is 33.6 times higher in those with a score 5 than the children with a score 0 to 4.

## Conclusion

Non-*E. coli* uropathogen growth, the presence of hydronephrosis and especially UTD-P3 dilatation on USG, presence of multiple scars and decreased relative function on DMSA scintigraphy were found to be associated with severe VUR. Our predicting model of severe VUR based on these variables appears to be effective.

## Supplementary Information

Below is the link to the electronic supplementary material.

Graphical abstract (PPTX 72.3 KB)

## Data Availability

The collected data are not available as open data.
