# Supplementary material for: Prediction model for severe vesicoureteral reflux in children with urinary tract infection and/or hydronephrosis
Source: Pediatr Nephrol. 2025 Jan 21;40(6):1975–82. doi: 10.1007/s00467-025-06668-7 (PMC12031799; doi:10.1007/s00467-025-06668-7)
Supplement: Supplementary file 1 — Graphical abstract (PPTX 72.3 KB) [file 467_2025_6668_MOESM1_ESM.pptx]

## Slide 1
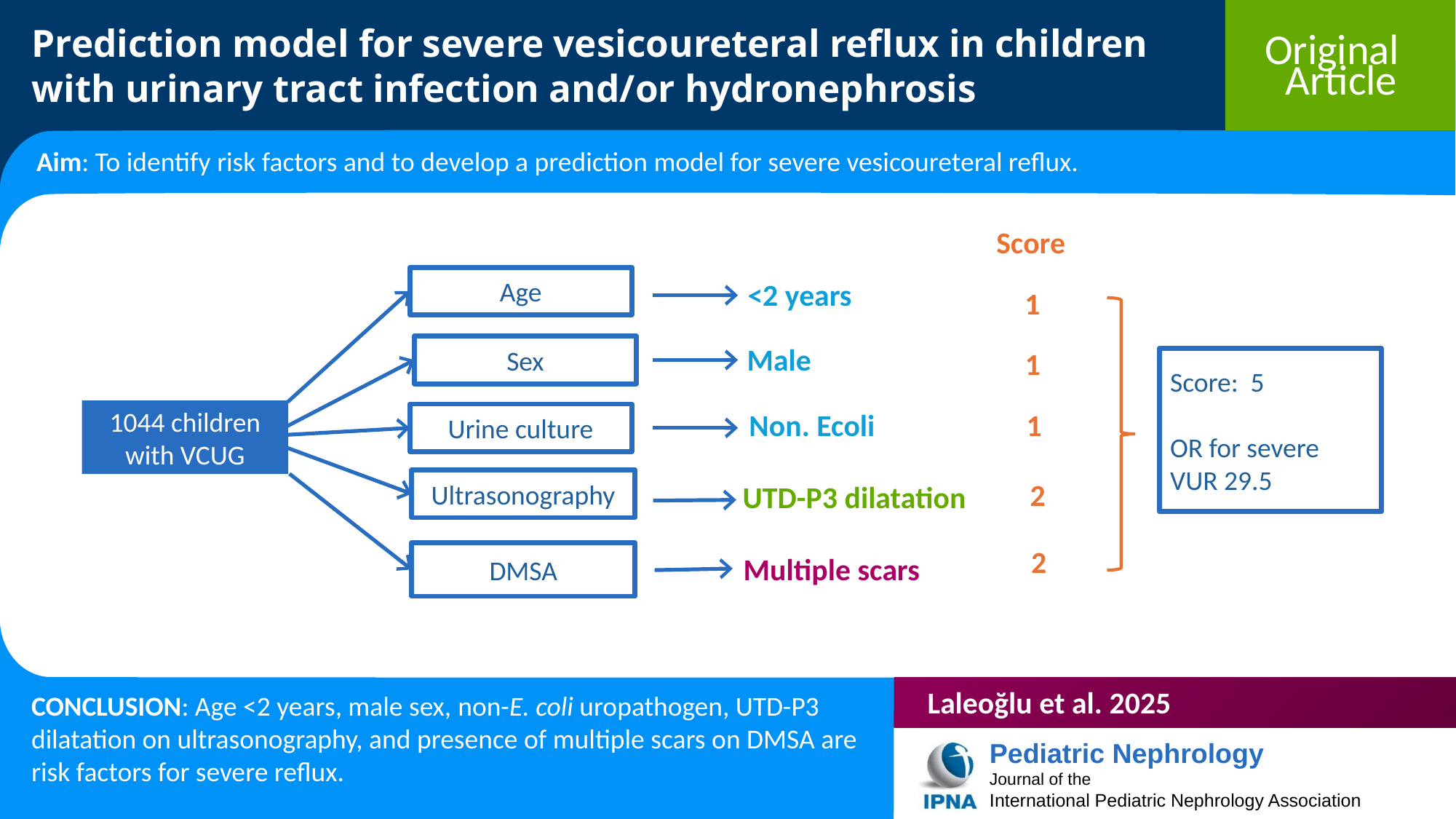

Prediction model for severe vesicoureteral reflux in children with urinary tract infection and/or hydronephrosis
Aim: To identify risk factors and to develop a prediction model for severe vesicoureteral reflux.
Score
Age
<2 years
1
Male
Sex
1
1
1044 children with VCUG
Non. Ecoli
Urine culture
Ultrasonography
2
UTD-P3 dilatation
2
DMSA
Multiple scars
Laleoğlu et al. 2025
CONCLUSION: Age <2 years, male sex, non-E. coli uropathogen, UTD-P3 dilatation on ultrasonography, and presence of multiple scars on DMSA are risk factors for severe reflux.
